# Simultaneous Increase of Solvent Flux and Rejection of Thin-Film Composite Membranes by Incorporation of Dopamine-Modified Mesoporous Silica

**DOI:** 10.1021/acsomega.1c01966

**Published:** 2021-06-08

**Authors:** Qianqian Tian, Wenrui Mu, Fei Shi, Yifan Li

**Affiliations:** School of Chemical Engineering, Zhengzhou University, Zhengzhou 450001, P. R. China

## Abstract

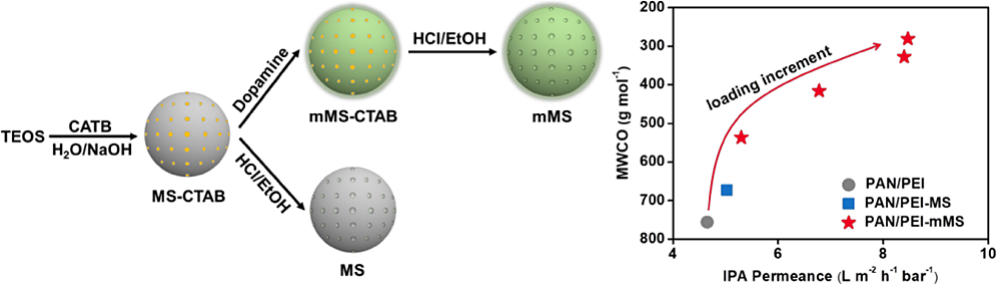

Thin-film nanocomposite membranes have shown great promise in organic solvent nanofiltration. However, it is challenging to acquire high permeation flux without severe swelling, which might do harm to rejection and long-term stability. In this study, we introduced dopamine-modified mesoporous silica nanoparticles into the polyamide (PA) matrix via interfacial polymerization to fabricate a series of thin-film nanocomposite membranes. By using polyethyleneimine (PEI) as the aqueous monomer, the modified nanoparticles are designed to be cross-linked within the PA network, which allows the penetration of PEI into the mesopores, and therefore, the membranes show better resistance to solvent-induced swelling and pressure-induced densification. More importantly, the mesopores of nanoparticles provide additional fast channels for solvents, resulting in an unusual enhancement of solvent flux under reduced membrane swelling. Along with the permeation flux, the rejection performance of the nanocomposite membranes is simultaneously improved, thanks to the controlled swelling arising from the strong interfacial adhesion. Thin-film nanocomposite membranes with optimal filler concentration exhibit a high isopropanol permeance of 8.47 L m^–2^ h^–1^ bar^–1^ as well as a quite low-molecular-weight cutoff of 281 Da.

## 1 Introduction

Polymer-based thin-film composite membranes have long been the major candidates for practical membrane separation because of the reliability of production and transportation of large-area membranes.^1−5^ The bottleneck of polymeric membranes is the so-called “trade-off” between permeability and selectivity, which is typically observed for gas separation, pervaporation, desalination, and organic solvent nanofiltration.^6−8^ In particular, for liquid separation, the solvent-induced membrane swelling usually results in enhanced permeation flux and decreased selectivity or rejection.^9−11^ Considering that excessive swelling would increase the concerns on membrane stability, the acquisition of high flux at high degree of membrane swelling is not reasonable. Boosting the solvent permeance of polymeric membranes by swelling-independent mechanisms is necessary to overcome the trade-off hurdle for liquid separation.^12−14^

Incorporation of permeable inorganic fillers into the polymeric thin film is acknowledged as a promising strategy to enhance the membrane permeance.^15,16^ Since inorganic materials often show much better resistance to swelling compared to polymeric materials, the transport pathways provided by inorganic fillers can efficiently facilitate solvent transport without the aid of membrane swelling.^17−20^ For instance, Xu et al.^21^ synthesized novel β-CD-enhanced ZIF-8 nanoparticles and incorporated into the PA layer. The resultant membrane (PPA2505) showed a higher solvent permeance with excellent antiswelling properties. Concretely, the area swelling ratio of the PPA2505 membrane in THF decreased by 58% in comparison with the pristine PA membrane, which is attributed to the structure reinforcement effect of the MPD-TMC-β-CD@ZIF-8 cross-linked network. However, the introduction of fillers is known to disrupt the polymer chain stacking efficiency, which might decrease the membrane stability against swelling if there are insufficient interactions between polymers and fillers. One should be cautious when selecting the fillers containing organic moieties, like metal−organic frameworks, in cases when they show instability such as polymers do.^22,23^ In addition, the pore size of the majority of fillers falls into the range of micropores (<2 nm).^24,25^ This design is meant to maintain high rejection of the relatively large solutes, but it may not be the best choice according to the interfacial morphology theory.^26,27^ Since strong interactions are required at the interface to control the swelling, the interface is expected to show the “chain rigidification” morphology, which would decrease the permeability of fillers. Also, the inevitable pore blockage at the interface is known to further restrict the full use of the filler channels.^28^ As suggested by Ismail and coworkers, the “ideal” case of the interface morphology—as desired for the simultaneous enhancement of permeability and selectivity—for large-pore fillers falls in the range of the “chain rigidification” or “pore blockage” region for typical microporous fillers.^26^ Although such a theory is developed based on gas permeation data, its availability in liquid separation is also worth evaluating.

Herein, nanosized mesoporous silica (MS) modified by dopamine was introduced for the fabrication of thin-film composite membranes. Silica is very stable in almost all kinds of solvents, and the employment of dopamine modification can guarantee adequate cross-linking among polymers and fillers. As such, we can better evaluate the contribution of porosity of fillers to solvent permeance. The mesopores were designed to see whether the solvent permeation results show the “ideal” case of the interface morphology as expected. Linear polyethyleneimine (PEI) was selected as the aqueous monomer, which was reported to be able to enter the mesopores of silica^29−31^ and therefore can further form a stable organic–inorganic network. The results show that the as-prepared membranes did become more robust and permeable, and a simultaneous increase in selectivity was observed from the decrease in the molecular weight cutoff.

## 2 Results and Discussion

### 2.1 Characterization of MS Nanoparticles

**Figure 1:**
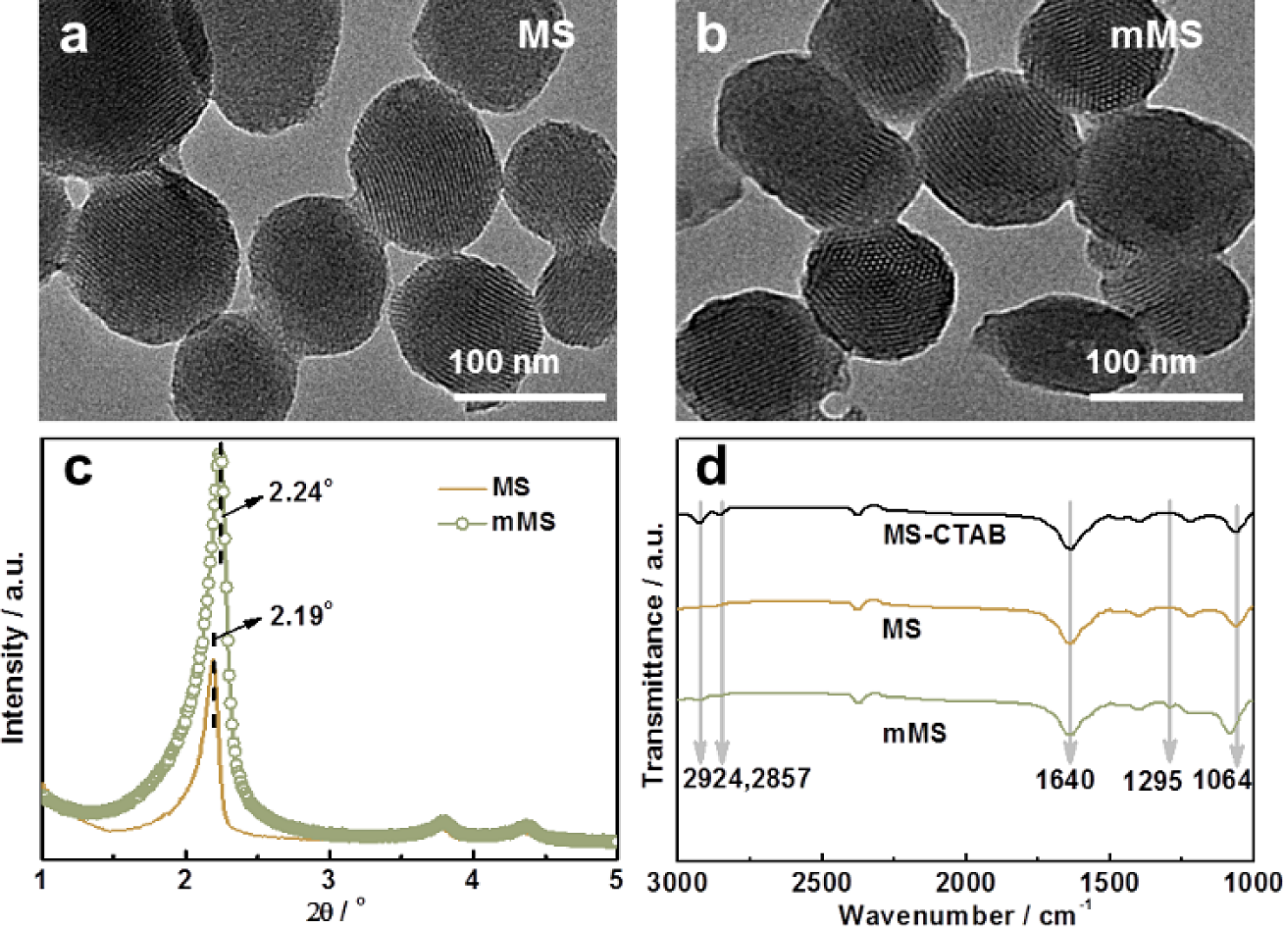
TEM images (a,b), XRD patterns (c), and FTIR spectra (d) of MS and mMS.

Figure 1a,b shows the transmission electron microscopy (TEM) images of the MS nanoparticles, which show sphere-like shapes and a diameter of about 100 nm. The parallel one-dimensional channels can be clearly seen throughout the particles and arranged in a hexagonal configuration,^32^ corresponding to the morphologies of the assembling micelles from CATB templates. The particles in Figure 1b (modified MS (mMS)) do not show the distinguished outer layer compared to Figure 1a (MS), indicative of the low amount of deposited polydopamine and the loss of polydopamine during the template removal procedure. The XRD patterns (Figure 1c) show the strong diffraction peak at 2θ = 2.19° for MS, corresponding to an interplanar spacing of 3.66 nm. For mMS, this spacing slightly decreases to 3.55 nm, indicating that the dopamine modification process had little impact on the removal of *N*-cetyltrimethylammonium bromide (CTAB) and the mesoporous structures. Fourier transform infrared (FTIR) spectra (Figure 1d) show the representative band of Si–O–Si at 1064 cm^–1^ for the asymmetric stretching and the band at 1640 cm^–1^ for the bending vibration of −O–H. The bands at 2924 and 2857 cm^–1^ in the spectrum of MS-CTAB are assigned to the −C–H stretching vibration, corresponding to the long aliphatic chain of CTAB. For MS and mMS, these characteristic bands are not detected, indicating the highly efficient removal of CTAB. For mMS, the appearance of the band at 1295 cm^–1^, assigned to the phenolic C–O stretching vibration, demonstrates the presence of dopamine at the surface. Moreover, the band at 1064 cm^–1^ for MS-CTAB and MS shifts is found to shift to a higher wavenumber in the spectrum of mMS, hinting that dopamine enables the formation of hydrogen bonds between the oxygen atoms of Si–O–Si and the hydrogen atoms of hydroxyl/amino groups.

**Figure 2:**
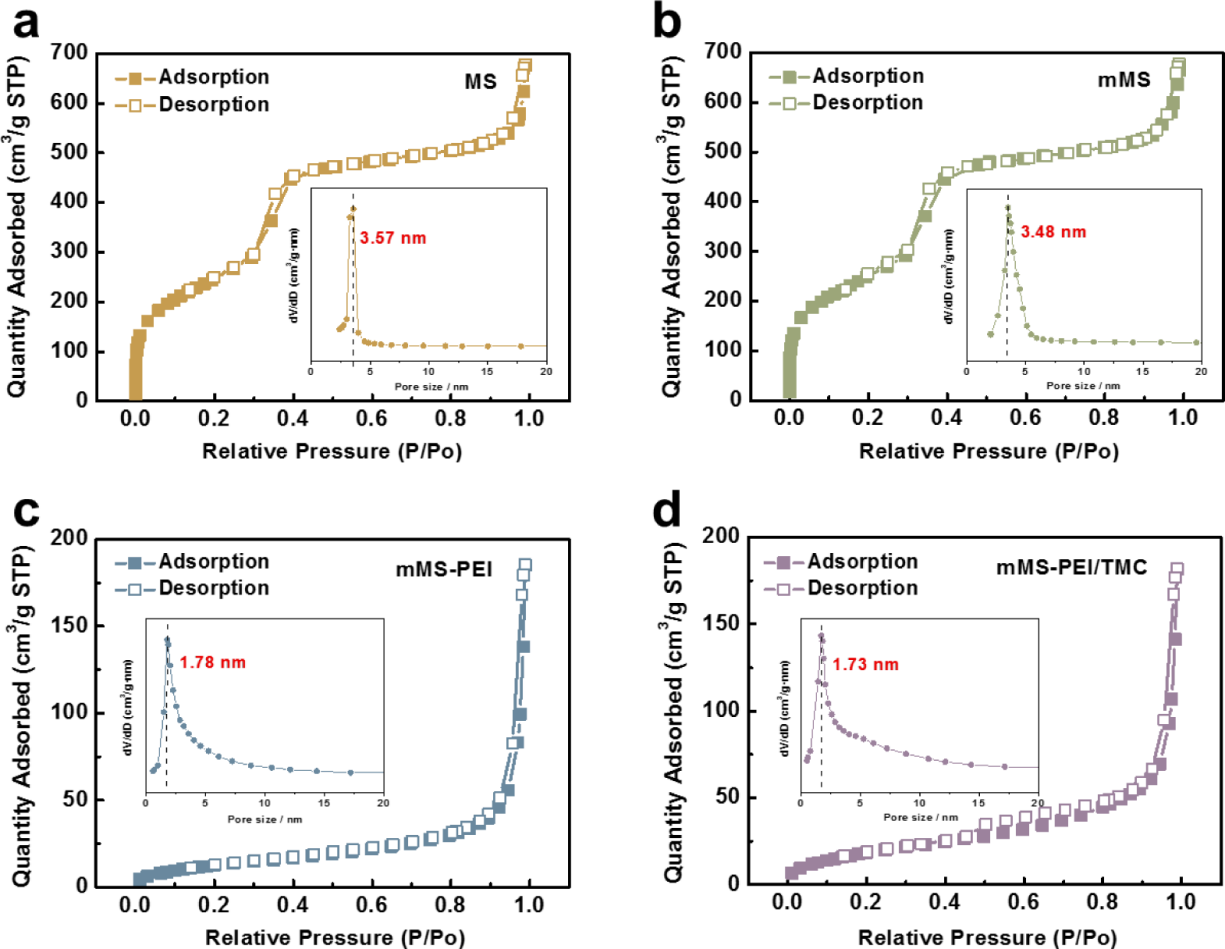
Nitrogen adsorption–desorption isotherms and pore size distribution of MS (a), mMS (b), mMS-PEI (c), and mMS-PEI/TMC (d).

To characterize the changes in the pore size and specific surface area of MS and mMS, the BET characterization was carried out (Figure 2 and Table 1). The isotherms indicate that MS is a typical mesoporous material with uniform mesoporous channels and a relatively narrow pore-size distribution (Figure 2a). mMS exhibits homologous isotherms, as shown in Figure 2b, which also affirms the mesoporous structure of mMS. Meanwhile, the BET results reflect the pore sizes of MS and mMS to be 3.57 and 3.48 nm, respectively, which can also confirm that the dopamine modification process does not affect the removal of CTAB and the original mesoporous structure of mMS. These results are in agreement with the XRD results to a great extent. Figure 2c,d displays typical type I isotherms for mMS-PEI and mMS-PEI/TMC, and the corresponding FTIR spectra are presented in Figure S1. After treating with the aqueous monomer and organic monomer, the pore size and specific surface area decrease from 3.48 nm and 747 m^2^ g^–1^ for mMS to 1.73 nm and 286 m^2^ g^–1^ for mMS-PEI/TMC, respectively, suggesting that the pores of mMS cannot be totally filled in the IP process.

**Table 1:** Surface Area Calculated by the BET Method for Different MS-Based Samples

samples	MS	mMS	mMS/PEI	mMS/PEI-TMC
*S*_BET_ (m^2^/g)	909	747	362	286

### 2.2 Characterization of Membranes

**Figure 3:**
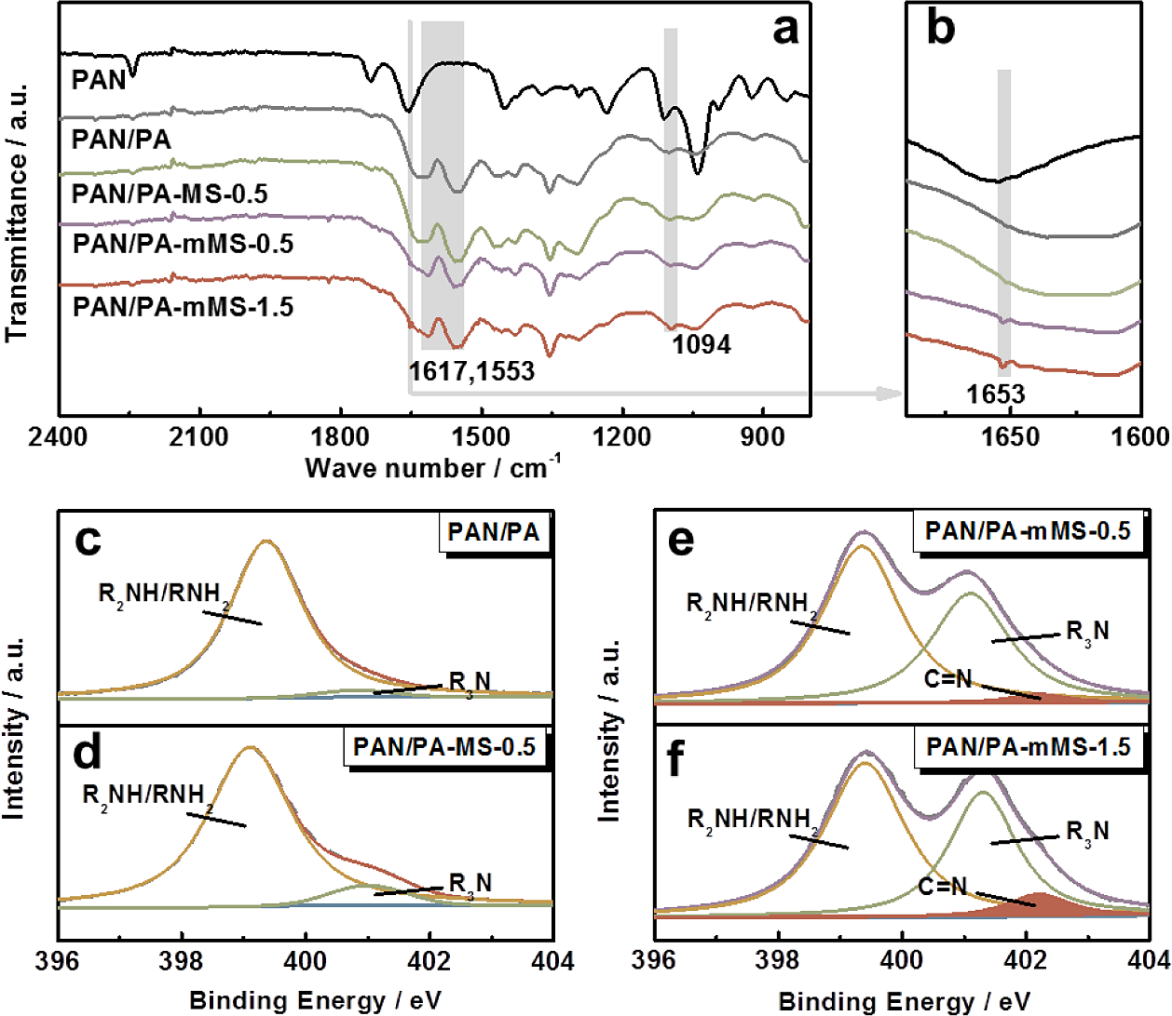
FTIR spectra of the PAN substrate and PA composite membranes (a,b) and high-resolution XPS N 1s spectra of PAN/PA (c), PAN/PA-MS-0.5 (d), PAN/PA-mMS-0.5 (e), and PAN/PA-mMS-1.5 (f) membranes.

The FTIR spectra of the membranes are presented in Figure 3a,b to analyze the chemical structures and the corresponding reactions during interfacial polymerization (IP). The appearance of the characteristic band of −C≡N at 2244 cm^–1^ for polyacrylonitrile (PAN) and its disappearance for the TFC membranes demonstrate the complete coverage of a film on the PAN support layer after polymerization. The bands at 1617 and 1553 cm^–1^ are assigned to C=O stretching and N—H deformation vibration, respectively, supporting the formation of amide bonds. After the incorporation of fillers, the spectra almost remain unchanged because of the relatively low filler content. However, a weak shoulder peak at 1653 cm^–1^ is observed for PAN/PA-mMS-0.5 and PAN/PA-mMS-1.5 but not observed for PAN/PA-MS-0.5, indicating the occurrence of the Schiff base reaction between dopamine and PEI and the formation of the C=N bond. This evidence demonstrates that mMS particles have been covalently connected with the polyamide (PA) networks via dopamine.

The XPS characterization of PAN/PA, PAN/PA-MS-0.5, PAN/PA-mMS-0.5, and PAN/PA-mMS-1.5 membranes was conducted, and their high-resolution XPS N 1s spectra are exhibited in Figure 3c–f. For the PAN/PA and PAN/PA-MS-0.5 membranes (Figure 3c,d), the peaks at 399.3 and 401.2 eV derive from amide or residual amine groups in the PA layer because of the interface polymerization reaction. However, compared with PAN/PA and PAN/PA-MS-0.5 membranes, a new peak appears at 402.2 eV for both PAN/PA-mMS-0.5 and PAN/PA-mMS-1.5 membranes (Figure 3e,f), which is attributed to C=N. These results indicate that the Schiff base reaction occurred between mMS and PEI. We can find that the peak becomes more intense with an increase in the filler content, which suggests that the Schiff base also becomes stronger. The results agree well with the FTIR.

The morphologies of the membranes are shown in Figures S2 and 4. PAN/PA shows a relatively smooth surface with nanosized granules. Such spotted structures might result from the diffusion-controlled interfacial reaction since PEI as a macromolecule in the aqueous phase cannot afford rapid diffuse like amines with small molecular weight. The high PEI concentration in aqueous phase solution (up to 8 wt %)—which facilitates the reaction but retards diffusion—gives it more opportunities to obtain hierarchical structures far from the thermodynamic equilibrium. After the incorporation of the fillers, the membrane surface becomes rougher with microsized protuberances connected with each other. Considering the particle size of MS and mMS, these protuberances are thought to reflect the morphologies of PA, which can be formed as a result of the interruption of the PA chain by fillers. Since PEI can partially or completely diffuse into the mesopores when preparing the aqueous monomer solution, the stretching and packing of polymer chains would be far from equilibrium, and local stress is expected to generate, which can further cause heterostructures at a larger scale. With mMS as the filler, the strong interactions between the filler and PA matrix lead to a further increase in the local stress associated with the protuberances, and the surface roughness (74.0 nm) is higher than that of the MS-filled membrane (66.2 nm). If we take a closer look at the surface of PAN/PA-mMS-1.5 membrane, we can also find submicron-sized granules and threads. The cross-sectional scanning electron microscopy (SEM) images of PAN/PA and PAN/PA-mMS-1.5 membranes are shown in Figures S3 and 5b, which reflect the actual thickness of membranes. The thicknesses of PAN/PA and the PAN/PA-mMS-1.5 membrane are about 572 and 685 nm, respectively. When the tip was carefully controlled to scan the surface without contacting the protuberances by atomic force microscopy (AFM), we found that the actual thickness of the PA layer is about 700 nm (Figure 5b,c), which is close to the maximum roughness and is several times larger than the particle size and the average roughness. The particles are therefore envisaged to distribute within the whole layer, rather than merely at the surface or throughout the film thickness. Such a mixed matrix structure technologically needs the relatively thick active layer to maintain defect-free for the fabrication of large-area membranes.

**Figure 4:**
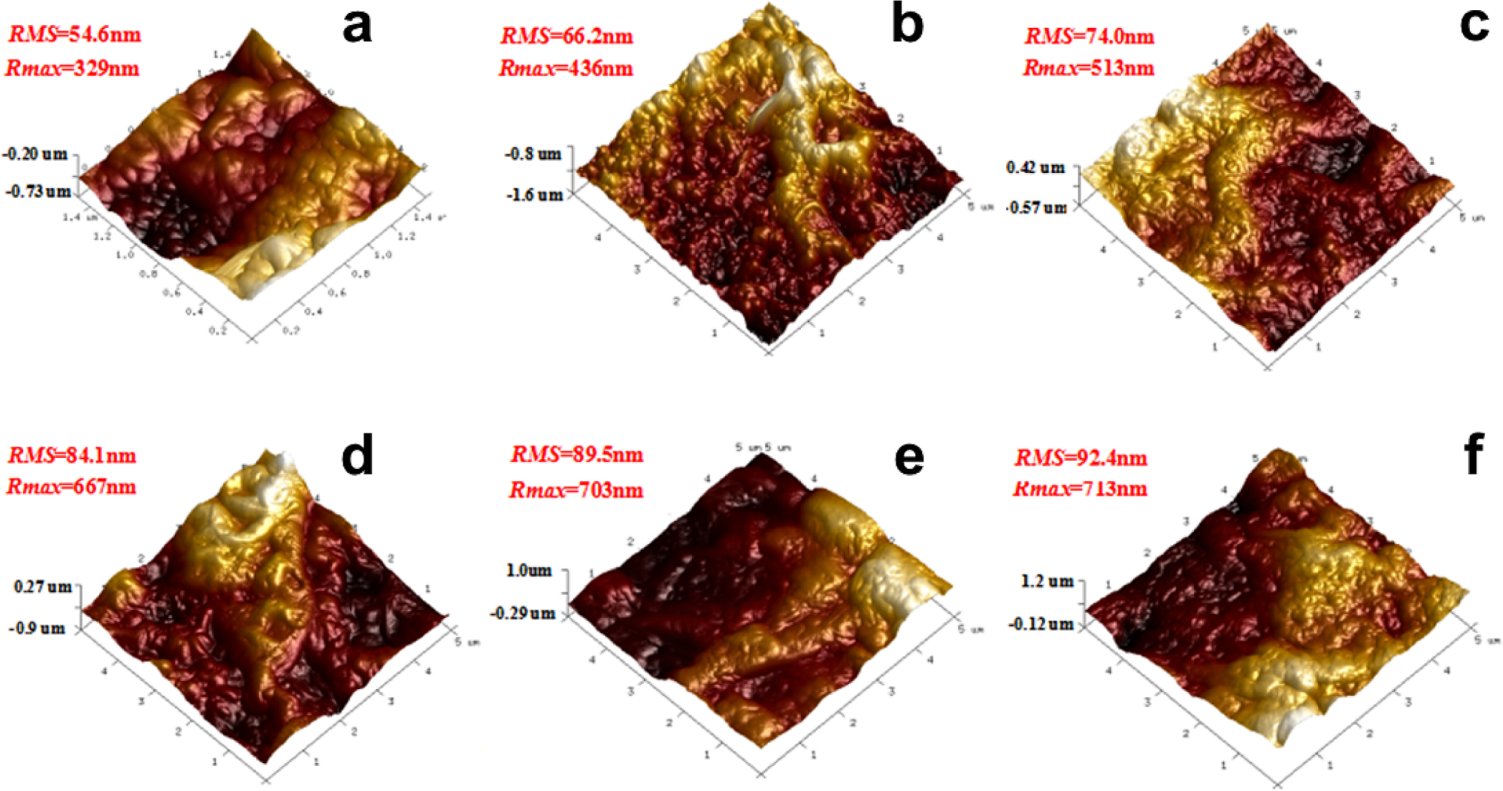
3D AFM images and roughness of PAN/PA (a), PAN/PA-MS-0.5 (b), and PAN/PA-mMS-*X* (*X* = 0.5, 1.0, 1.5, and 2.0) (c–f) membranes.

**Figure 5:**
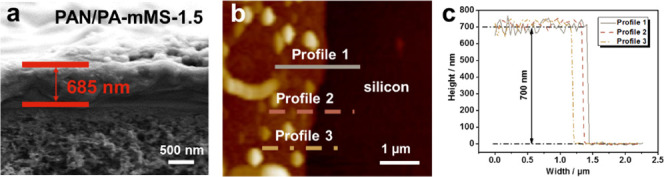
Cross-sectional SEM image (a), AFM height image (b), and the corresponding height profiles (c) of the PAN/PA-mMS-1.5 membrane.

**Figure 6:**
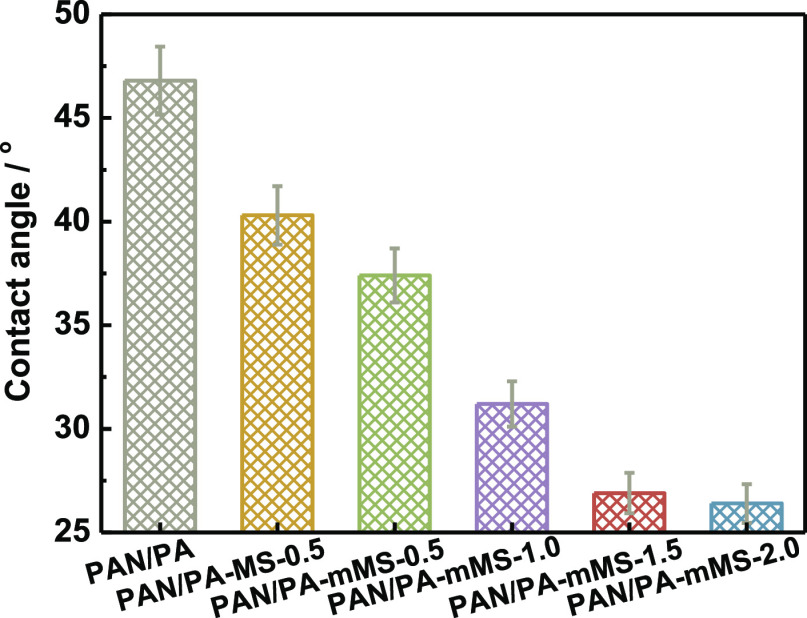
Contact angles of PA composite membranes.

The water contact angle data shown in Figure 6 are used to characterize the surface hydrophilicity of the membranes. The control membrane, PAN/PA, shows a contact angle of about 47°, demonstrating a rather hydrophilic surface. The embedding of fillers (both MS and mMS) leads to a decrease in the contact angle—that is, improvement of hydrophilicity. With an increase in the mMS content, the contact angle further decreases down to about 27°. Such a decrease in the contact angle is usually elucidated by the introduction of hydrophilic groups (with a higher polarity or area density) to the membrane surface or an increase in the surface roughness. Herein, both the factors play pivotal roles. On the one hand, the trend of the contact angle variation corresponds well with that of the surface roughness. On the other hand, in our previous study, the employment of polydopamine nanoparticles as fillers caused a higher contact angle of up to 56° at similar surface roughness (80 nm).^33^ Since polydopamine particles were synthesized in strongly alkaline solution for a long period (5 h) and hence underwent deep oxidation, most phenolic groups might be converted into quinone groups for further cross-linking. In this study, the dopamine modification process was controlled within 1 h under mild conditions (pH = 8.5). In the literature, dopamine coating onto the porous support under such a pH value was reported to obtain the most hydrophilic membrane surface, compared to other pH values adopted.^34^

### 2.3 Solvent Uptake and Swelling Resistance of Membranes

**Figure 7:**
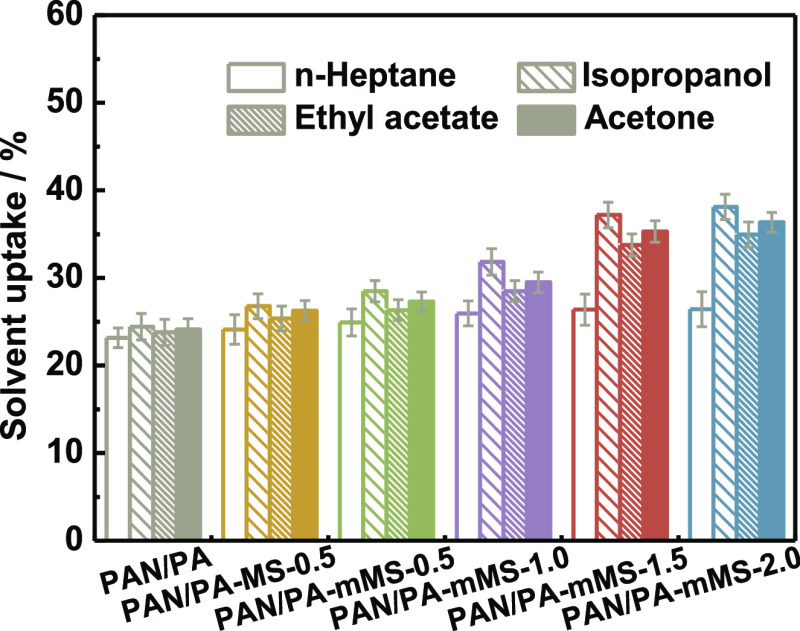
Solvent uptake of PA composite membranes for *n*-heptane, ethyl acetate, isopropanol, and acetone.

**Figure 8:**
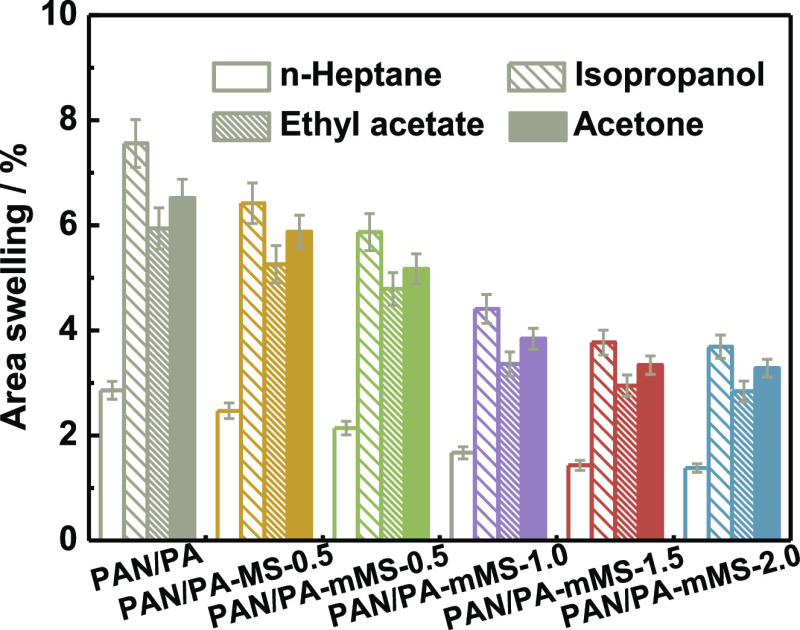
Area swelling of PA composite membranes for *n*-heptane, ethyl acetate, isopropanol, and acetone.

Four typical solvents, *n*-heptane, isopropanol, ethyl acetate, and acetone, are used to probe the solvent uptake (Figure 7) and swelling resistance (Figure 8) of membranes. The porous PAN substrate displays almost the same uptake (∼20%) for all of the solvents. The same case is observed for PAN/PA with only a slight increase in the solvent uptake. After the incorporation of the fillers, the uptake of three polar solvents exhibits a substantial increase (up to ∼35% for PAN/PA-mMS-1.5 and PAN/PA-mMS-2.0), which is consistent with an increase in surface roughness. However, the change in the membrane area swelling shows the opposite trend—that is, solvent uptake increases with the reduction in the swelling degree. This unusual fact not only reveals the good swelling resistance of the membranes ascribed to the robust hybrid network but also provides important clues to understand the mechanism of the solvent uptake in the membranes. On account of the low filler content and the thin active layer, the large extent of solvent uptake increment is predominantly attributed to the trapped solvent within the microsized cavities or “valleys” beneath the external surface, which restricts the removal of some nonadsorbed solvents by capillary force and friction drag. By comparison, the uptake of the nonpolar solvent, *n*-heptane, remains almost unchanged, which illustrates that the nonpolar solvent has little chance to wet the intrinsically hydrophilic membrane surface and is therefore difficult to enter the active layer. The similar trend of the membrane-dependent area swelling to that of polar solvents illustrates that the antiswelling properties are solvent-independent, which might have great promise in organic solvent nanofiltration. Nevertheless, the solvent uptake still depends on the solvent type. The uptake of the four solvents follows the sequence of polarity: isopropanol > acetone > acetate > *n*-heptane, which demonstrates that the membrane does possess highly hydrophilic characteristics.

### 2.4 Organic Solvent Nanofiltration Performance of Membranes

**Figure 9:**
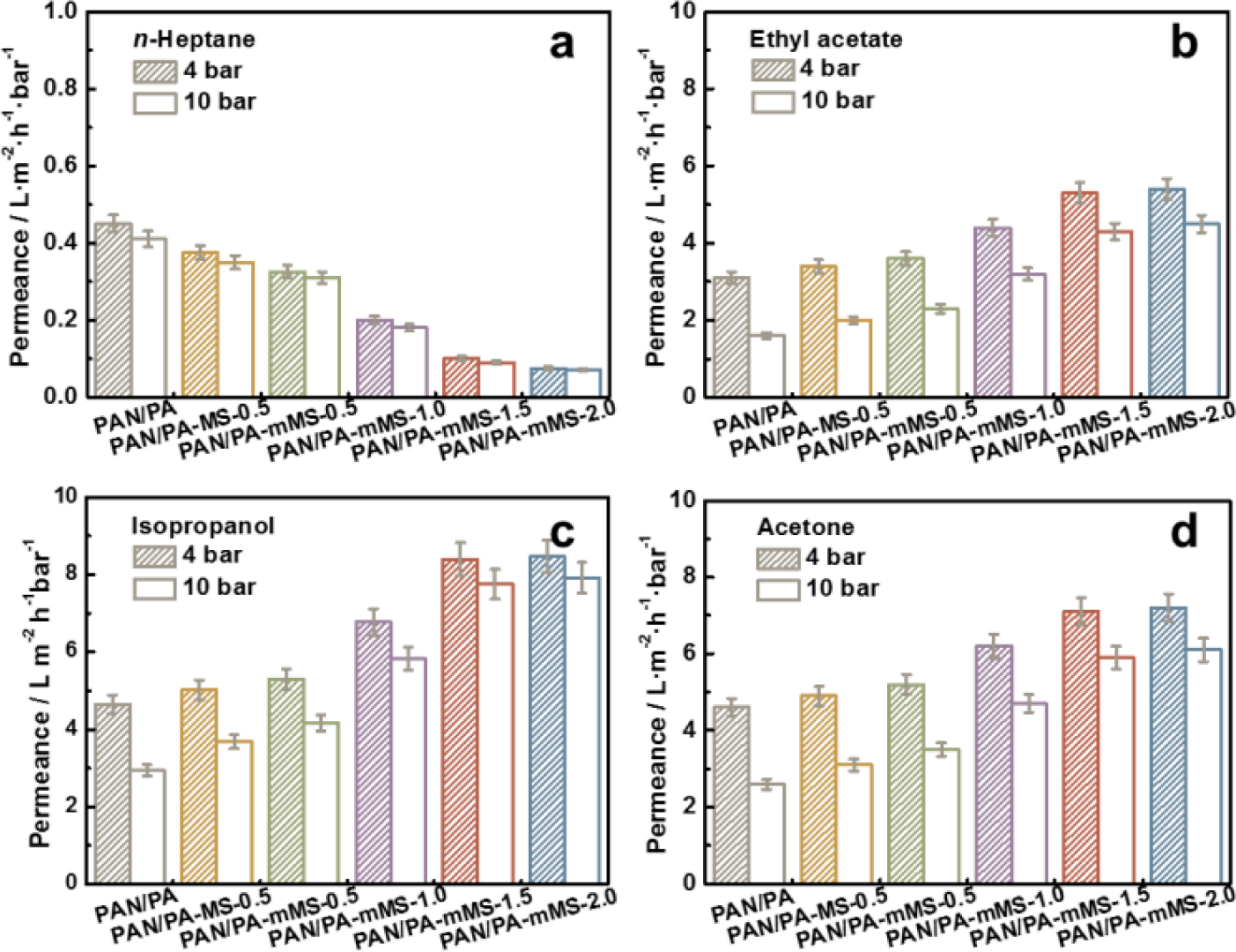
Permeance of PA composite membranes for *n*-heptane (a), ethyl acetate (b), isopropanol (c), and acetone (d).

**Scheme 1:**
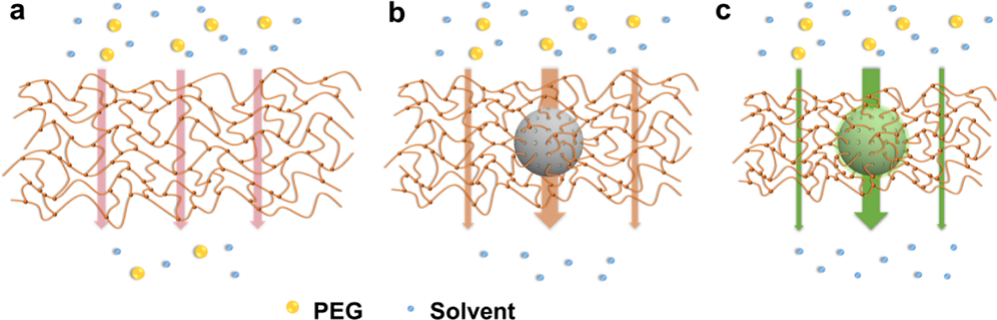
Schematic Illustration of Swelling and the Solvent Transport Ability of the Pristine PA Membrane (a), MS-Filled Membrane (b), and mMS-Filled Membrane (c)

The permeation flux of the four solvents through each membrane can be found in Figure 9. The flux of the four solvents follows the sequence of polarity: isopropanol > acetone > ethyl acetate > *n*-heptane, as the solvent uptake data shown in Figure 7. In accordance with the hydrophilicity and the swelling data, *n*-heptane records the lowest flux among the four solvents, and the doping of fillers further decreases the flux value by about 80% on both 4 and 10 bar. The flux values of three polar solvents are 1 magnitude higher than that of *n*-heptane, indicating that the membranes only afford fast solvent permeation upon good wetting. Thus, the membranes have the potential to separate polar solvents from aliphatic solvents. However, a substantial increase in the permeation of flux with mMS incorporation can be observed for the three polar solvents on both 4 and 10 bar, which is not consistent with the swelling data. That is, the highest flux is recorded by the membrane with the lowest swelling degree. This unusual result can be understood from two aspects: (i) membrane-to-solvent affinity, considering that the highest flux still corresponds to the highest solvent uptake and (ii) diffusion resistance, as the incorporated mesopores can serve as highways for solvent permeation. In this sense, the enhancement of the solvent flux benefits from the increment in affinity and the diffusion pathways within membranes, rather than the loosening of the PA network (shown in Scheme 1). Another interesting phenomenon is that the permeance—a pressure-normalized flux—at 10 bar becomes more close to the data obtained at 4 bar with an increase in the filler content. It is known that the polymeric membrane conventionally becomes compact and less permeable under elevated pressures.

As shown in Figure 9b–d, for the PAN/PA control membrane, the permeance at 10 bar is only about half of the value at 4 bar. For PAN/PA-mMS-2.0, the difference is significantly reduced. In particular, the isopropanol permeance at 10 bar even reaches 94% of the data at 4 bar. It is reasonable to hypothesize that the rigid inorganic fillers within the membranes prevent the membrane from excessive compression under high pressure.

**Figure 10:**
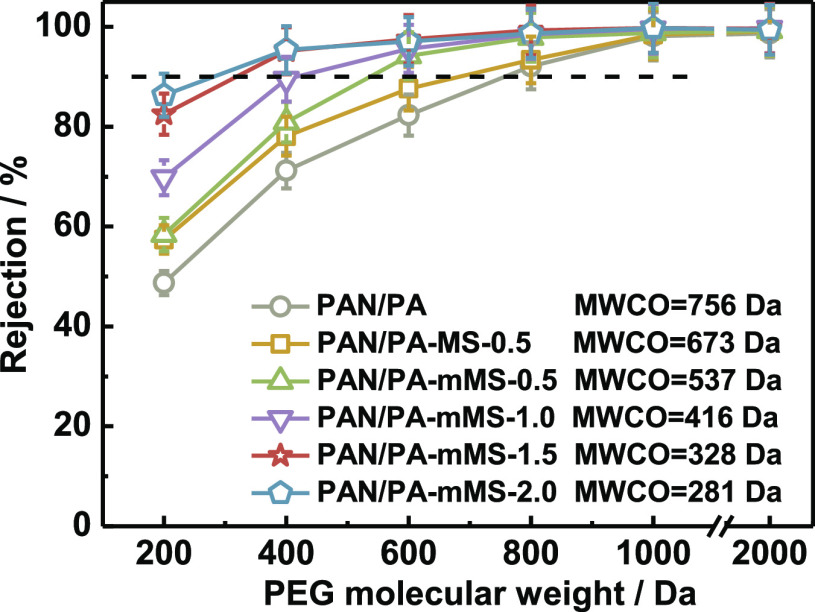
Retention curves of the membranes with isopropanol as the solvent at 10 bar.

**Figure 11:**
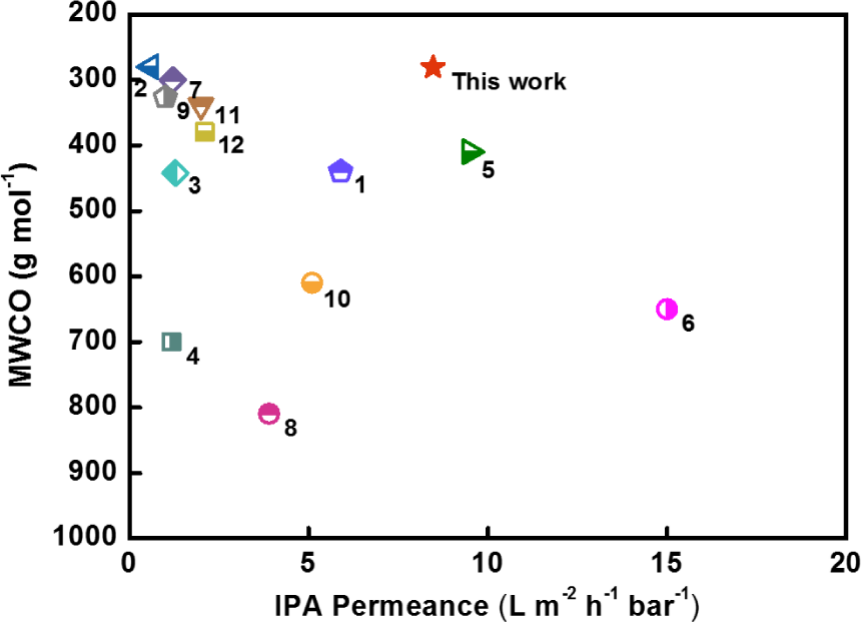
Comparison of the performance of the membrane in this work with other polymer-based membranes on MWCO–permeance plot (1, X-PBI;^37^ 2, TFC_0.025–0.25/0.75/20_;^38^ 3, S4-N-BAPP-20;^39^ 4, PAR@mBHPF0.2%;^40^ 5, MPCM;^41^ 6, PA-PAN;^42^ 7, GQD1/TMC;^43^ 8, PA/cGO/cross-linked PI;^44^ 9, Noria + TPC;^45^ 10, m-XDA/TMC-2/0.1;^46^ 11, sPPSU-C;^47^ 12, ANF.^48^

Considering that the membranes have resolved the trade-off between the solvent flux and swelling resistance, they also have great promise to overcome the trade-off hurdle between the solvent flux and rejection. As shown in Figure 10, the sequence of MWCO for the membranes shows the opposite trend to that of flux: PAN/PA > PAN/PA-MS > PAN/PA-mMS, and the MWCO further decreases with an increase in mMS loading. This result is in good accordance with the swelling data shown in Figure 8, demonstrating that the incorporated fillers can improve the rejection properties of the membrane by controlling the swelling degree. In addition, the thermodynamics and kinetics of reaction monomers may be affected in the IP process because of the incorporation of fillers, resulting in a higher cross-linking degree and smaller pore size.^35,36^ This effect also favors the enhancement of the rejection ability and becomes more distinct with an increase in filler loading. In this way, the simultaneous increase in the flux and rejection is achieved. The optimal membrane shows a rather high isopropanol flux (8.47 L m^–2^ h^–1^ bar^–1^) with a low MWCO (281 Da), which is much better than those reported in our previous study, where polydopamine nanoparticle was selected as the filler (isopropanol flux of 2.0 L m^–2^ h^–1^ bar^–1^ and MWCO of 380 Da),^33^ indicating the important contributions from the MS and the corresponding beneficial interfacial morphology—rigidification and pore blockage. This performance also outperforms the majority of polymer-based membranes on the MWCO–permeance plot (Figure 11).

**Figure 12:**
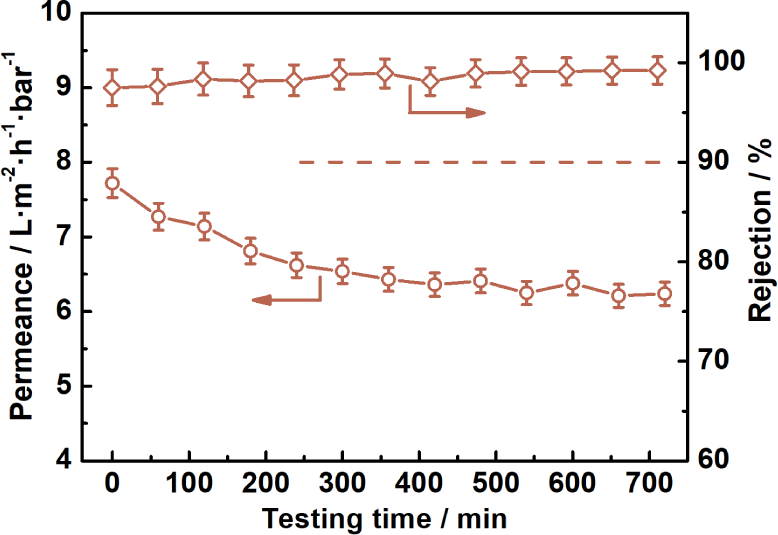
Long-term operational stability of the PAN/PA-mMS-1.5 membrane.

With both the nanofiltration performance and fabrication reliability considered, PAN/PA-mMS-1.5 was selected to evaluate the long-term operational stability, which is crucial for any membrane to be used for practical applications. The membrane was first immersed in isopropanol for a week, and then, the permeance and the rejection of PEG1000 (under 10 bar) were collected every 60 min within the whole testing period (720 min). As shown in Figure 12, the flux of isopropanol decreases from 7.72 to 6.25 L m^–2^ h^–1^ bar^–1^ with a reduction of 19% within the initial 540 min and remains almost constant during the rest of the testing time. During the same period, the rejection ratio first increases from 97.5% to 99.2% and then remains steady. Such results are likely ascribed to the compaction of the membrane and polyethylene glycol (PEG)-induced “fouling” on the membrane surface, as shown in Figure S4.^37,43^ At the end of the test, isopropanol flux and rejection are 6.24 L m^–2^ h^–1^ bar^–1^ and 99.3%, respectively, demonstrating the excellent operation stability that benefits from the antiswelling architecture of the membrane.

## 3 Experiment

### 3.1 Materials

The PAN ultrafiltration membrane (MWCO of 100 kDa) was supplied by Zhongkeruiyang Membrane Engineering & Technology Co., Ltd. Trimesoyl chloride (TMC), CTAB, and PEI (*M*_w_ of 20 kDa) were obtained from Aladdin Chemical Co., Ltd. PEG (*M*_w_ of 200–2000 Da) was supplied by Alfa Aesar Chemical Co., Ltd. Dopamine hydrochloride, tris(hydroxymethyl)aminomethane, and tetraethyl orthosilicate (TEOS) were obtained from Yuancheng Technology Development Co., Ltd. Organic solvents (ethanol, ethyl acetate, isopropanol, acetone, *n*-heptane, and *n*-hexane) were supplied by Tianjin Kermel Chemistry Co., Ltd. Hydrochloric acid (HCl) and sodium carbonate (Na_2_CO_3_) were obtained from Kewei Chemistry Co., Ltd. All materials were used without any further purification. Deionized (DI) water was used throughout the whole experiment.

### 3.2 Synthesis of MS nanoparticles

The MS nanoparticles with a diameter of about 100 nm were synthesized as follows. First, 0.8 g of CTAB was dissolved in a mixture containing 480 mL of DI water and 3.5 mL of NaOH solution (2 mol L^–1^). Then, 5.0 mL of TEOS was quickly added, and the solution was vigorously stirred at 80 °C for 3 h. The resultant product (MS-CTAB) was separated by filtration, washing, and drying overnight. The 1.5 g of structure-template CTAB was removed by refluxing in a mixed solution of ethanol (160 mL) and HCl (9 mL) for 48 h at 80 °C. The template-removed MS nanoparticles were separated and obtained after filtration, washing, and drying overnight.

### 3.3 Synthesis of mMS nanoparticles

**Scheme 2:**
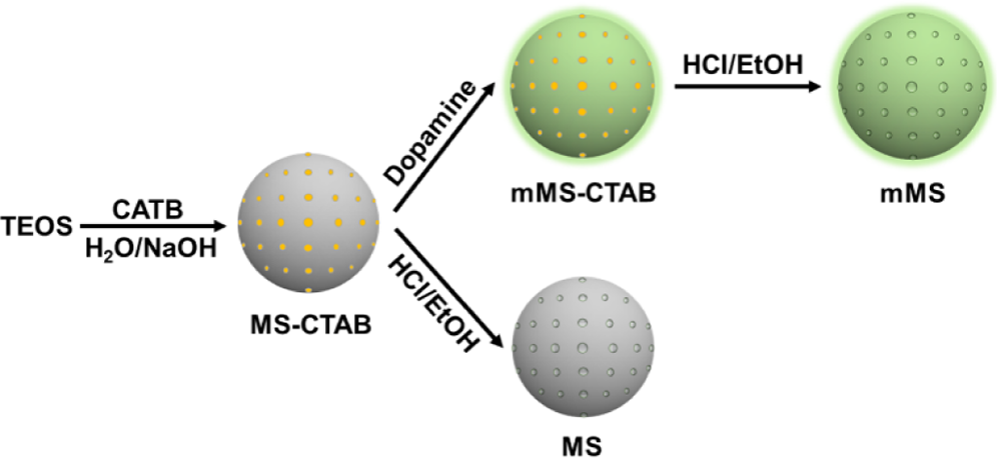
Synthesis of MS and mMS

MS-CTAB (1 g) prepared by the method described in Section 2.2 was dispersed in dopamine solution (1 g L^–1^), and the solution was treated with ultrasound for 30 min. The resultant (mMS-CTAB) was separated by filtration, washing, and drying overnight. The 1.5 g of structure-template CTAB was also removed by refluxing in a mixed solution of ethanol (160 mL) and HCl (9 mL) for 48 h at 80 °C. In this step, the pH value is as low as 2.5; therefore, the polydopamine oligomers that are not strongly attached at the MS surface would also be removed. It is important to note that the remaining dopamine does not have to fully cover the MS surface, according to its role at the polymer–filler interface of the membrane. The template-removed mMS nanoparticles were separated and obtained after filtration, washing, and drying overnight. The synthesis procedure of MS and mMS is illustrated in Scheme 2.

### 3.4 Membrane Preparation

PA composite membranes were prepared by IP. We investigated the effect of the aqueous monomer concentration and polymerization reaction time on the membrane nanofiltration performance (Figure S5), and the optimal conditions in membrane preparation is monomer concentration of 8 wt % and a reaction time of 10 min. Concretely, PEI as the monomer of the aqueous phase was dissolved in water with the concentration of 8 wt %. mMS nanoparticles were also added in the aqueous phase and treated with ultrasound for 1 h. TMC as the monomer of the organic phase was dissolved in hexane with the concentration of 2 wt %.

First, the PAN substrates were immersed in water for 30 min. Aqueous phase solution was initially introduced into the surface of PAN substrates and was allowed to stand for 10 min. Then, the excess aqueous phase was removed, and the soaked membranes were dried at room temperature until no free liquid was observed. After that, organic phase was added to the surface of membranes. After the reaction time of 10 min, the extra solution was removed. The membranes were kept in a vacuum oven at 25 °C for 1 h and then at 60 °C for 12 h. The as-prepared membranes were identified as PAN/PEI-mMS-*X*, where *X* means the mass percentage content of mMS for PEI. In addition, PA composite membranes containing MS named PAN/PEI-MS-*X* were also fabricated with the same method for comparison.

### 3.5 Characterization

The nanoparticles and membranes morphologies were characterized by TEM (Tecnai G2 F20, FEI, USA) and SEM (Auriga FIB SEM, Zeiss, Germany). The surface structure, roughness, and thickness of membranes were estimated by AFM (Bruker Dimension FastScan). The pore size of MS nanoparticles was detected by X-ray diffraction (XRD, RigakuD/-max2500 v/Pc). The chemical compositions of nanoparticles and membranes were detected by FTIR (Nicolet MAGNA-IR560 Instrument). The water contact angles of the membranes were measured on FACE (model OCA 25, Germany) at room temperature.

### 3.6 Nanofiltration Performance of the Membranes

A homemade dead-end unit cell with a volume of 200 mL (typically the feed volume is about 150 mL) was employed to measure the solvent permeance and PEG rejection of the membranes. The effective permeation area of the cell was 18.2 cm^2^. The organic solvent and PEG solution (500 mg L^–1^) were utilized to evaluate the membrane performance in a N_2_-pressurized cell. The membranes were immersed in solvent for several hours in advance to ensure adsorption equilibrium. Before the measurement of the nanofiltration performance at 4 or 10 bar, the membranes were precompacted with solvent or PEG solution at 4.5 or 10.5 bar for about 30 min to obtain a steady permeation state. Subsequently, the pressure was turned to 4 or 10 bar for the further test. Batch addition of the feed solution was employed to minimize the effect of the solute concentration in the retentate. Once the permeate volume reached 10 mL, the equivalent volume of fresh feed solution would be added to the feed cell. The long-term operation (720 min at 10 bar) was also conducted in this way. Concretely, the permeance (*P*, L m^–2^ h^–1^ bar^–1^) was calculated through *P* = *V*/(Δ*p* × *A* × *t*) with the permeate volume (*V*, L), pressure difference (Δ*p*, bar), membrane area (*A*, m^2^), and time (*t*, h). PEG solution was poured into the cell equipped with magnetic stirring (500 rpm) to estimate the rejection of membranes. The PEG concentration in the permeate was measured by the UV–vis method to acquire the rejection. The rejection (*R*, %) was measured by *R* (%) = (1 – *C*_p_/*C*_f_) × 100 with the concentration of the permeate solution (*C*_p_) and pristine feed (*C*_f_). Notably, the PEG concentration in the permeate was measured to obtain the mean value after reaching the adsorption equilibrium, and the dynamic adsorption data of the membranes are provided for comparison (Figure S6 and Table S1). All of the solvent permeation and nanofiltration experiments were performed three times.

## 4 Conclusions

In this study, thin-film nanocomposite membranes were designed and fabricated by incorporating dopamine-modified MS nanoparticles into a PA matrix prepared by IP. PEI as a macromolecular aqueous monomer enables the conjunction of organic and inorganic domains by entering the mesopores or reacting with the dopamine moieties on the filler surface. Such a robust hybrid network provided three benefits: (i) the decrease in swelling, which improved the rejection performance and long-term operation stability; (ii) the increase in surface roughness (up to ∼90 nm) and hydrophilicity, which provided more surface cavities for polar solvent retention; and (iii) the increase in mechanical stability, which prevented the membrane from excessive compaction under elevated pressure. Moreover, the mesopores further caused a decrease in the diffusion resistance within the membrane. Owing to these aforementioned benefits, the hybrid membrane showed a simultaneous increase in the solvent flux and rejection, supporting that the ideal interfacial morphology for mesoporous fillers is rigidification or pore blockage. This finding hints that the revised morphological diagram proposed by Ismail’s group from gas permeation data is also available in organic solvent nanofiltration. Considering the optimal performance (isopropanol permeance of 8.47 L m^–2^ h^–1^ bar^–1^ and a MWCO of 281 Da) was recorded by a membrane with moderate thickness (∼700 nm), we believe that the membranes have great promise in large-area production and commercial application.

## Supporting Information Available

Nanofiltration performance of the PA/PAN-MS-0.5 composite membrane as a function of PEI concentration and polymerization reaction time; the adsorption results of PAN and composite membranes for PEG400; FT-IR spectra of MS-based samples; surface SEM images of composite membranes; cross-sectional SEM image of PAN/PA membranes; and the antifouling ability of the PAN/PA-mMS-1.5 membrane (PDF)
