# Supplementary material for: Simultaneous Increase of Solvent Flux and Rejection of Thin-Film Composite Membranes by Incorporation of Dopamine-Modified Mesoporous Silica
Source: ACS Omega. 2021 Jun 8;6(24):16241–50. doi: 10.1021/acsomega.1c01966 (PMC8223411; doi:10.1021/acsomega.1c01966)
Supplement: Supplementary file 1 — ao1c01966_si_001.pdf [file ao1c01966_si_001.pdf]

# Supporting information

## Simultaneous increase of solvent flux and rejection of thin-film composite membranes by incorporation of dopamine-modified mesoporous silica

Qianqian Tian, Wenrui Mu, Fei Shi, Yifan Li*

School of Chemical Engineering, Zhengzhou University, Zhengzhou 450001, P. R. China.

Corresponding author:

*E-mail: yf_li@zzu.edu.cn

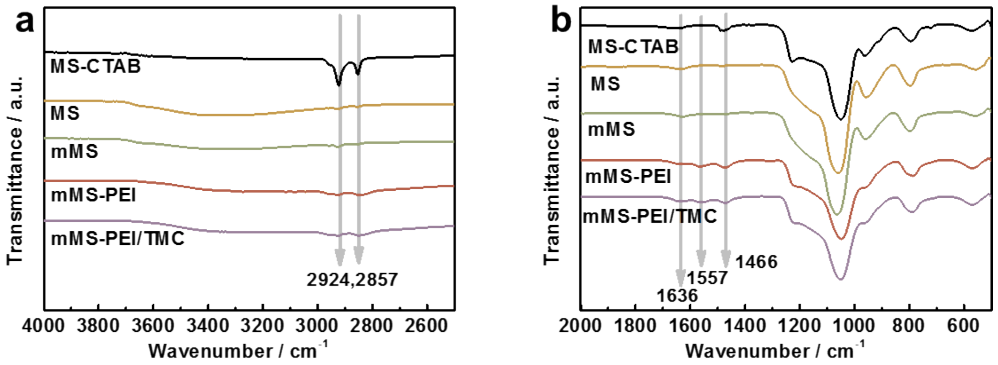


**Figure S1.** FT-IR spectra of MS-based samples at high wave numbers(a); at low wave numbers(b).

FT-IR spectra of MS-based samples are presented in Figure S1 to analyze the chemical structures. After treated with aqueous monomer and organic monomer, the peaks at 1557 and 1466 $cm^{-1}$ appear in the spectrum of mMS-PEI, which is assigned to -NH bending vibration and $-CH_2$ stretching vibration. For mMS-PEI, the stronger peak at 1636 $cm^{-1}$ corresponding to the C=O stretching vibration in amide group confirms the the occurrence of interfacial polymerization.

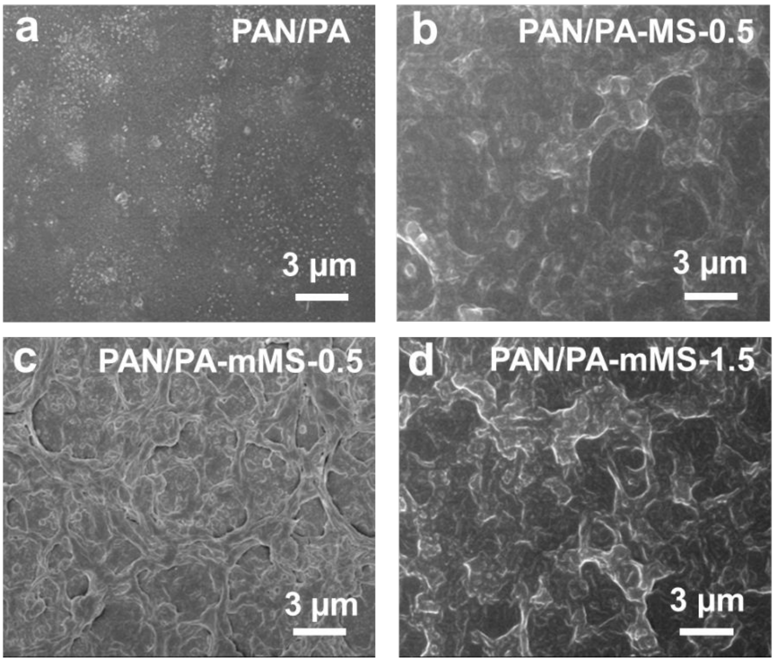


**Figure S2.** SEM images of PAN/PA(a), PAN/PA-MS-0.5(b), PAN/PA-mMS-0.5(c) and PAN/PA-mMS-1.5(d) membranes.

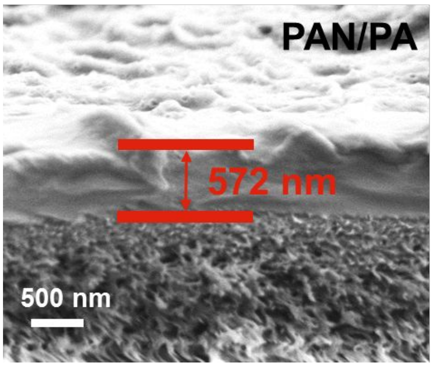


**Figure S3.** Cross-sectional SEM image of PAN/PA membrane

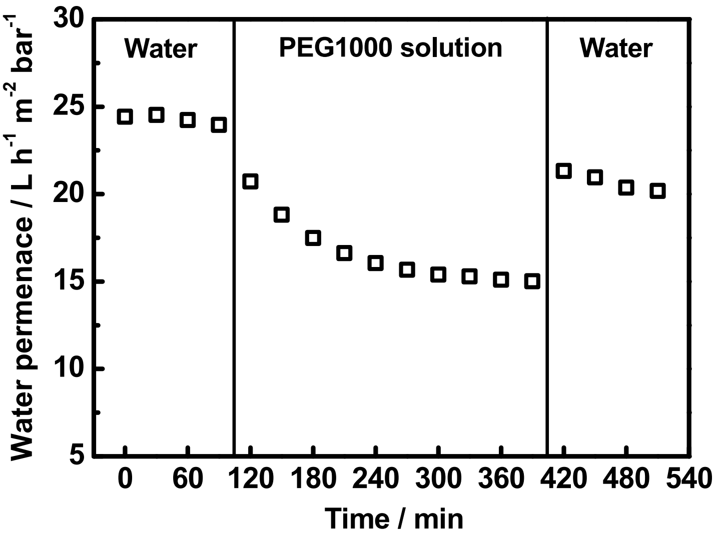


**Figure S4.** The anti-fouling ability of PAN/PA-mMS-1.5 membrane

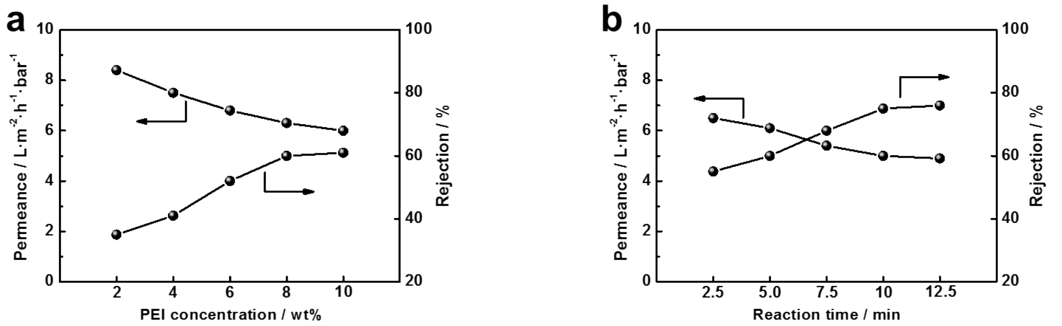


**Figure S5.** Isopropanol permeance and PEG-400 rejection of PA/PAN-MS-0.5 composite membrane as a function of PEI concentration(a) and polymerization reaction time(b).

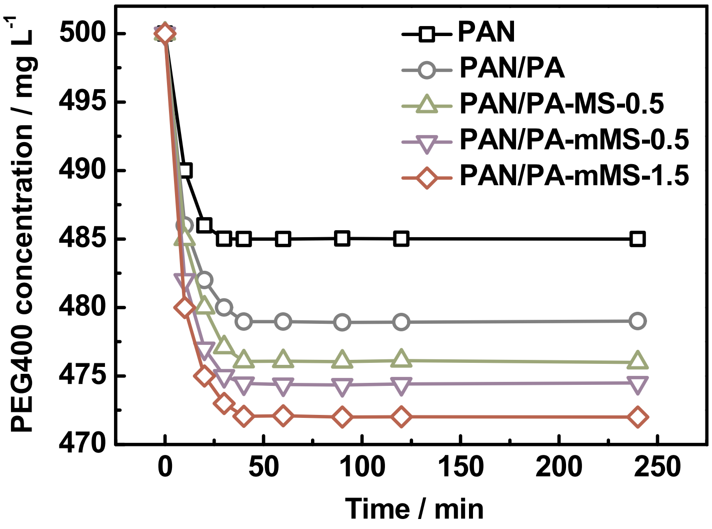


**Figure S6.** Effect of adsorption time on the PEG400 concentration.

**Table S1.** Equilibrium adsorption rate of PEG400 for PA composite membranes.

| **Samples** | PAN/PA | PAN/PA-MS-0.5 | PAN/PA-mMS-0.5 | PAN/PA-mMS-1.5 |
|---|---|---|---|---|
| **Adsorption rate(%)** | 4.2 | 4.8 | 5.1 | 5.6 |
